# Optical imaging of voltage and calcium in isolated hearts: Linking spatiotemporal heterogeneities and ventricular fibrillation initiation

**DOI:** 10.1371/journal.pone.0215951

**Published:** 2019-05-14

**Authors:** Ismael Hernández-Romero, María S. Guillem, Carlos Figuera, Felipe Atienza, Francisco Fernández-Avilés, Andreu M. Climent

**Affiliations:** 1 Department of Signal Theory and Communications, Universidad Rey Juan Carlos, Madrid, Spain; 2 Hospital General Universitario Gregorio Marañón, Instituto de Investigación Sanitaria Gregorio Marañón (IISGM), Madrid, Spain; 3 ITACA, Universitat Politècnica de Valencia, Valencia, Spain; 4 CIBERCV, Centro de Investigación Biomédica en Red de Enfermedades Cardiovasculares, Madrid, Spain; 5 Facultad de Medicina, Universidad Complutense, Madrid, Spain; University of Minnesota, UNITED STATES

## Abstract

**Background:**

Alternans have been associated with the development of ventricular fibrillation and its control has been proposed as antiarrhythmic strategy. However, cardiac arrhythmias are a spatiotemporal phenomenon in which multiple factors are involved (e.g. calcium and voltage spatial alternans or heterogeneous conduction velocity) and how an antiarrhythmic drug modifies these factors is poorly understood.

**Objective:**

The objective of the present study is to evaluate the relation between spatial electrophysiological properties (i.e. spatial discordant alternans and conduction velocity) and the induction of ventricular fibrillation (VF) when a calcium blocker is applied.

**Methods:**

The mechanisms of initiation of VF were studied by simultaneous epicardial voltage and calcium optical mapping in isolated rabbit hearts using an incremental fast pacing protocol. The additional value of analyzing spatial phenomena in the generation of unidirectional blocks and reentries as precursors of VF was depicted. Specifically, the role of action potential duration (APD), calcium transients (CaT), spatial alternans and conduction velocity in the initiation of VF was evaluated during basal conditions and after the administration of verapamil.

**Results:**

Our results enhance the relation between (1) calcium spatial alternans and (2) slow conduction velocities with the dynamic creation of unidirectional blocks that allowed the induction of VF. In fact, the administration of verapamil demonstrated that calcium and not voltage spatial alternans were the main responsible for VF induction.

**Conclusions:**

VF induction at high activation rates was linked with the concurrence of a low conduction velocity and high magnitude of calcium alternans, but not necessarily related with increases of APD. Verapamil can postpone the development of cardiac alternans and the apparition of ventricular arrhythmias.

## Introduction

The initiation of ventricular arrhythmias is promoted by the presence of (i) dynamic factors and (ii) a heterogeneous substrate, e.g. structurally abnormal hearts or myocardial ischemia, which allows the maintenance and perpetuation of the arrhythmia [1]. Dynamic factors are related to the ability of a region to show electrophysiological time variations between beats, associated with steep conduction velocity (CV) restitution [2,3] and instability of calcium cycling factors [4,5]. Spatial heterogeneities are due to gradients of expression levels of ion channel and transporter proteins [6] in healthy tissue [7,8], or caused by structural pathologies or ischemia [9], manifesting three-dimensional patterns of action potential durations (APD) and ionic concentration flows, as in intracellular calcium transients (CaT).

At high cardiac rates, heterogeneous cardiac substrate leads to an arrhythmogenic behavior and electric instability, which may manifest in the form of alternans. The presence of alternans is a biomarker commonly associated with the onset of ventricular fibrillation (VF) and sudden cardiac death in clinical practice [10]. Alternans are defined as oscillations in AP and CaT amplitude and duration between successive beats, which can produce two types of spatial patterns: (i) spatially discordant alternans (SDA), when alternans in adjacent regions occur in opposite phases, and (ii) spatially concordant alternans (SCA), if they occur in phase between the regions.

The presence of SDA is clinically relevant because the increase of heterogeneity of repolarization is assumed to be more arrhythmogenic [10,11]. Formation and progression of SDA have been closely linked to the nature of the cardiac substrate [4,12]. Moreover, some antiarrhythmic drugs have shown their effectiveness in reducing the incidence of VF by modifying these specific cardiac features [13–16]. However, the sequence of events and the multiple electrophysiological factors by which SDA can promote VF, and how these are modified by an antiarrhythmic treatment, have not been fully studied.

In this study, we use simultaneous optical mapping of transmembrane voltage and intracellular calcium concentration to analyze the role of these parameters on the onset of VF. More specifically, we studied the relation of reduced CVs and the spatial distribution and magnitude of alternans with the initiation of reentry and VF. In addition, these measurements were also compared with those under the administration of verapamil, an L-type calcium channel blocker which modulates the appearance of alternans and incidence of sudden cardiac death. Our main findings support that heterogeneous distribution of cardiac substrate and dynamic factors of calcium cycling at the organ level have a main role in the generation of reentry and VF, and that the therapeutic control of these factors can avoid the trigger of these events.

## Materials and methods

### Isolated heart preparation

Experiments were performed in five New Zealand rabbits at Hospital Gregorio Marañon in Madrid. This study was carried out in accordance with the recommendations of the directive 2010/63/EU on the protection of animals used for scientific purposes as well as the European and Spanish regulations on the subject, after the approval by the committee of ethics in animal experimentation of Hospital Gregorio Marañon. In particular, rabbit hearts were isolated by thoracotomy after general anesthesia using pentobarbital and immersed in cardioplegic solution at 4°C for their transport (in mM: 140 NaCl; 5.4 KCl; 1 MgCl_2_; 5 HEPES; 11 Glucose; 1.8 CaCl_2_ with a pH of 7.4). After removal of coronary blood, hearts were retrogradely perfused through the aorta using a cannula with a constant flow of modified Tyrode’s solution at 36.5°C (in mM: NaCl, 120, NaHCO_3_, 25, CaCl_2_, 1.8, KCl, 5.4, MgCl_2_, 1, glucose, 5.5, H_2_PO_4_H_2_O, 1.2, pH 7.4). The medium was continuously oxygenated by the bubbling of carbogen (95% oxygen / 5% carbon dioxide). All compounds were purchased from Sigma-Aldrich (Dorset, UK).

### Optical mapping system

**Fig 1:**
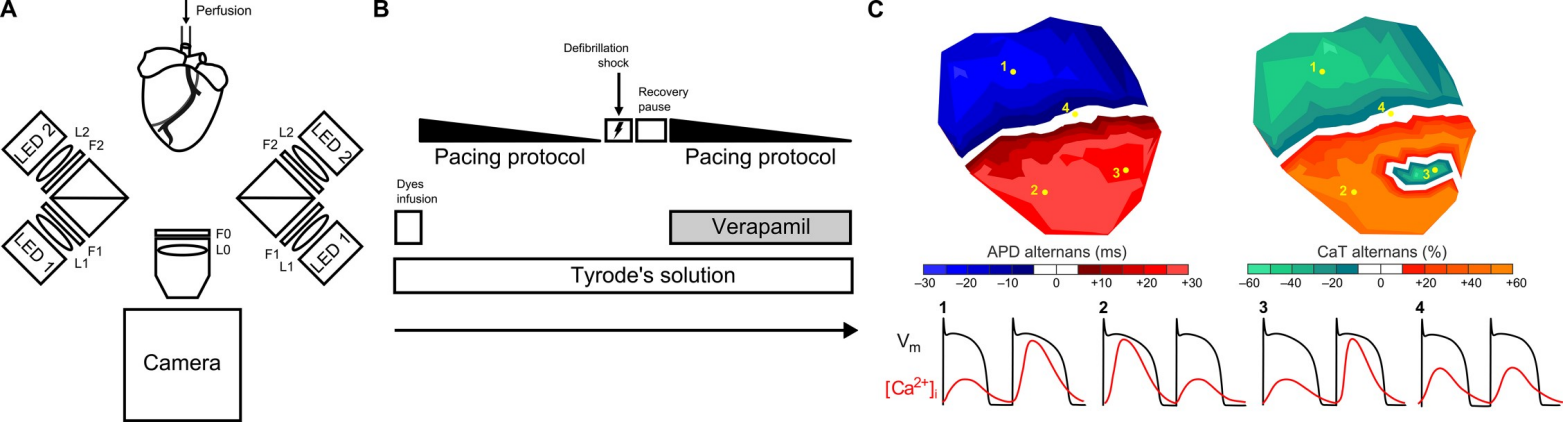
Experimental setup used. **(A)** Diagram of the optical mapping system. **(B)** Schematic diagram of the experimental protocol. **(C)** Representation of APD and CaT alternans maps and signals that compose them during a spatially discordant stage: (1) a short-long pattern with positive coupling, (2) a long-short pattern with positive coupling, (3) a negative coupling and (4) a nodal line region without alternans.

Hearts were infused with Di-4-ANBDQPQ voltage-sensitive dye (excitation/emission wavelength of ~640 nm/~750 nm) with an aortic bolus through the cannula of 20 μL at 35 mM (donated by Dr. Loew, University of Connecticut Health Center) for 5 minutes and 250 μL at 1 mM of Rhod2-AM calcium sensitive dye (excitation/emission wavelength of ~555 nm/~585 nm) (Biotium, Hayward, CA, USA) for 30 minutes, replacing the infusion with fresh Tyrode’s solution containing 10 mM of Blebbistatin (Biotium) [17]. Di-4-ANBDQPQ was excited by the use of a CBT-90-R (LED1; Peak Wavelength 628 nm, Luminus Devices, Billerica, MA, USA) LED light using an excitation-filter D640/20X (F1; Chroma Technology, Bellows Falls, VT, USA). Rhod-2AM was excited by a white light (LED2; LED-CBT-90-W65S-C11-LA100; Luminus Devices) using an excitation-filter S555/25X (F2; Chroma Technology) [18]. The lights were collimated with a flat-convex lens (L1 and L2; LA1951; Thorlabs, Newton, NJ, USA). The fluorescence emission of the dyes was recorded through a specifically designed multiband filter ET585/50-800/200M (F0; Chroma Technology) plus a long-pass filter with significant transmission at wavelengths >575 nm (F0; BLP01-561R-25; Semrock, Rochester, NY, USA), placed in front of the camera lens (L0, Navitar, Rochester, NY, USA) for adequate collection of fluorescence emission [18]. The voltage and calcium recordings were performed using a high-density optical mapping system based on an electron-multiplied charge-coupled device (Evolve 128 EMCCD, 128x128 pixels, 24-um-square pixels, 16 bit; Photometrics, Tucson, AZ, USA). The signal was digitized with a 16-bit analog/digital converter with a sampling frequency of 512 Hz. A diagram of the system is represented in Fig 1A.

### Experimental protocol

Ventricular pacing was performed with standard clinical catheters with platinum iridium bipolar electrodes (diameter, 0.125 mm; inter-electrode distance, 1 mm). Pacing (2-ms bipolar rectangular pulses, intensity of 1–2 V twice the diastolic threshold) was carried out with a home-made high pacing unit. Hearts were defibrillated with a biphasic pulse of 30 J on the epicardium. All hearts were defibrillated after the first shock. Each animal was defibrillated only once. VF inducibility protocol was repeated after infusion of verapamil (2μM, 16.7 mg/Kg heart weight), using an aortic bolus through the cannula. The concentration was chosen in the rank in which its electrophysiological effects were observable.

A pacing protocol was applied at increasing rates, starting with a pacing cycle length (PCL) of 350ms and gradually increasing the frequency until the onset of VF (i.e. 350, 250, 200, 190, 180, 170, 160, 155, 150, 145, 140, 135, 130, 125, 120, 115, 110, 105, 100, 95, 90, 85, 80ms). For each pacing rate, two consecutive movies of 5 seconds were recorded. Pacing protocol and movie recording was automatized to ensure the shortest duration of the experiment. In case VF was induced, pacing protocol was finalized and 6 movies of 10 seconds were recorded before the defibrillation. Total duration of the pacing protocol was less than 10 minutes. Schematic diagram of the experimental protocol is shown in Fig 1B.

### Voltage and calcium imaging processing

Optical mapping signals were analyzed by using our customized software designed in MATLAB (The MathWorks, Massachusetts, United States). Raw data was filtered to eliminate fluorescence noise, applying a spatial Gaussian filter (size 7 × 7 pixels) and a temporal Savitzky-Golay filter (size 15 samples). For each pixel, baseline was removed by subtracting the lower envelope of the signals. Finally, voltage and calcium signals of each pixel were normalized between 0 and 1. Pixels outside the area of cardiac tissue were automatically discarded applying a user-defined mask [19]. APDs were measured at 80% of repolarization. CaT magnitude was defined as the peak-to-peak amplitude of a calcium transient, which lies between 0 and 1 due to the pixel-by-pixel normalization.

### Identification of voltage and calcium spatial alternans

Spatial analysis of the distributions of alternans for voltage and calcium signals was independently performed as follows: APD alternans were defined as the APD difference between simultaneous activations (Eq 1) and the CaT alternans maps as the percentual difference in amplitude of calcium transients between consecutive (n and n + 1) activations in each pixel (Eq 2):

$$\Delta APD{\left(x,y\right)}_{n}=APD{\left(x,y\right)}_{n+1}-APD{\left(x,y\right)}_{n}$$ (1)

$$\Delta CaT{\left(x,y\right)}_{n}=\frac{CaT{\left(x,y\right)}_{n+1}-CaT{\left(x,y\right)}_{n}}{max(CaT{\left(x,y\right)}_{n+1},CaT{\left(x,y\right)}_{n})}$$ (2)

where n is a time interval between successive activation times and x, y are the pixel coordinates. Applied thresholds were defined according to already validated values [20,21]. Only differences in APD above 2 ms and CaT differences above 5% were considered. Alternans were defined as present only when the short-long-short variation was found for at least 6 consecutive beats.

In Fig 1C, a schematic illustration of spatial alternans is represented. The alternating phase was determined positive for long-short APDs (represented by red scale) and for high-low CaT pulses; and negative for short-long APDs and for low-high CaT pulses. The regions without alternans were defined as nodal lines and shown in white. Percentage of area covered by alternans was calculated by dividing the amount of pixels that displayed alternans by the total number of recorded pixels. Mean values (and standard deviation, SD) of APD, ΔAPD and ΔCaT parameters were computed for each recording. ΔAPD and ΔCaT areas were fitted to single exponential curves (Eq 3):

$$f(x)=ae^{bx}$$ (3)

### Identification of conduction velocities

CV was measured in each pixel of voltage optical mapping with a variation of the method presented by Bayly et al. [22]. Specifically, the workflow of CV estimation included: (1) the phase of the voltage signal for each pixel was calculated using the Hilbert transform [23], (2) isochronal maps were obtained by detecting 2π changes in the phase, (3) a subpixel edge detector was used to identify the normal vector of propagation of wavefronts [24]., 4) normal vectors of propagations were used to identify wavefront trajectories and 5) CVs were computed in steps of 5ms over each trajectory of each wavefront. Distributions of CV were represented within isochrones, denoted by isolines that connect all the pixels with the same CV value.

### Statistical analysis

T-student test was used to evaluate null hypothesis of differences between basal and treated conditions in all 5 animals. Specifically, APD, alternans and CV were compared during basal conditions and after the administration of verapamil. P-values lower than 0.05 were considered statistically significant. All measurements are presented as mean ± standard deviation.

## Results

### Evolution of electrophysiological properties prior to VF

In order to understand the relation between the dynamic changes of electrophysiological properties and the initiation of VF, we examined the rate-dependent distributions of APD, CaT alternans and CV with optical mapping for increasing pacing rates until the development of VF.

**Fig 2:**
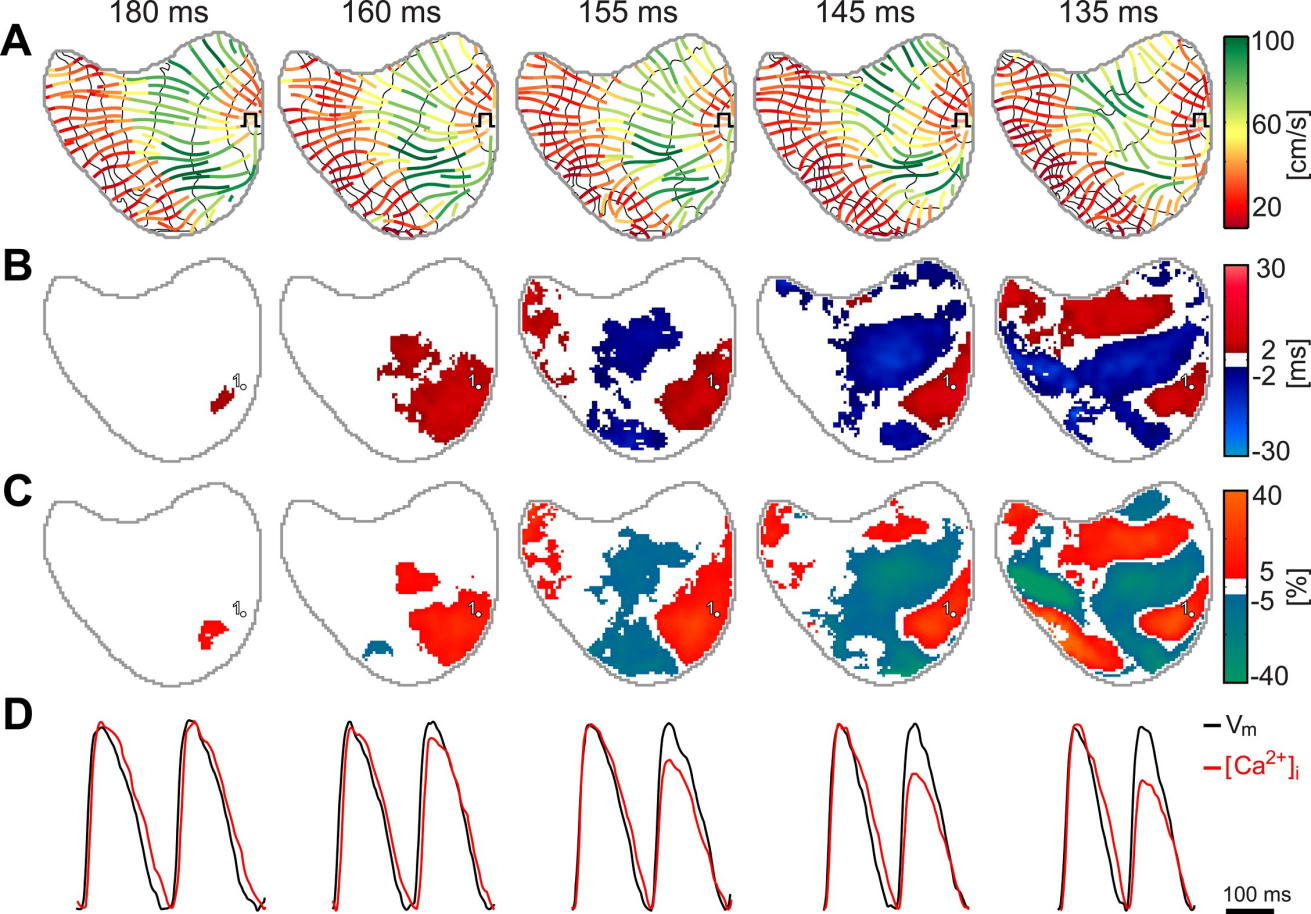
Rate-dependent development of electrophysiological features obtained at different pacing cycle length in basal conditions. **(A)** Distribution of local CV through the trajectory of propagation over isochrones fragmented in steps of 5 ms (black). **(B)** Maps of APD alternans obtained between successive wavefronts. **(C)** Maps of CaT alternans obtained between successive propagations. **(D)** Recordings of optical voltage and calcium signals.

In Fig 2, a representative example of the increasing complexity of spatial alternans (i.e. APD and CaT) and the time required for a signal to propagate across the epicardium at different pacing rates is shown. In Fig 2A, lines of propagation with a color scale representing the CV are depicted on top of isochrones emerging from the pacing point in the left ventricle. Notice the variation of CV during the propagation from the pacing point through the epicardium, forming a CV gradient with three different bands distributed along the propagation direction: 1) a slow conduction area close to the pacing point, 2) an intermediate band where CVs are higher than that in surrounding areas, and 3) a second distal region of slow conduction. Distribution of the three CV regions remained stable for all pacing rates, while mean local CV tends to decrease with increasing stimulation rates in all three regions.

Fig 2B and 2C show APD and CaT alternans maps respectively. The magnitude of both APD and CaT alternans and their spatial complexity are increased with the activation rate. Small isolated islets of alternans appear at PCL = 180 ms close to transition between slow and fast propagating regions. As the frequency increased, number and area of islets in the epicardial area showing alternans also increased. Notice that, whereas for PCL of 180 ms alternans were positive in all islets, an increase in the pacing rate to 160 ms resulted in an increment in the difference of CaT and the appearance of spatial alternans with positive and negative areas (i.e. SDA). Specifically, for longer stimulation intervals, regions with SDAs were only detectable in calcium, while after an increase in the pacing rate, SDAs were also detectable in voltage. As the rate increased, the area covered by alternans grows until it covers almost the entire epicardium. We did not observe any relation between the appearance or location of nodal lines and isochrones. Interestingly, the size of each spatial alternant islet was inversely related with the distance to the pacing point; whereas spatial alternant areas close to the pacing point were large and with well-defined nodal lines, spatial alternant areas distal from the pacing point presented irregular shapes and smaller areas.

### Mechanism of onset of VF

**Fig 3:**
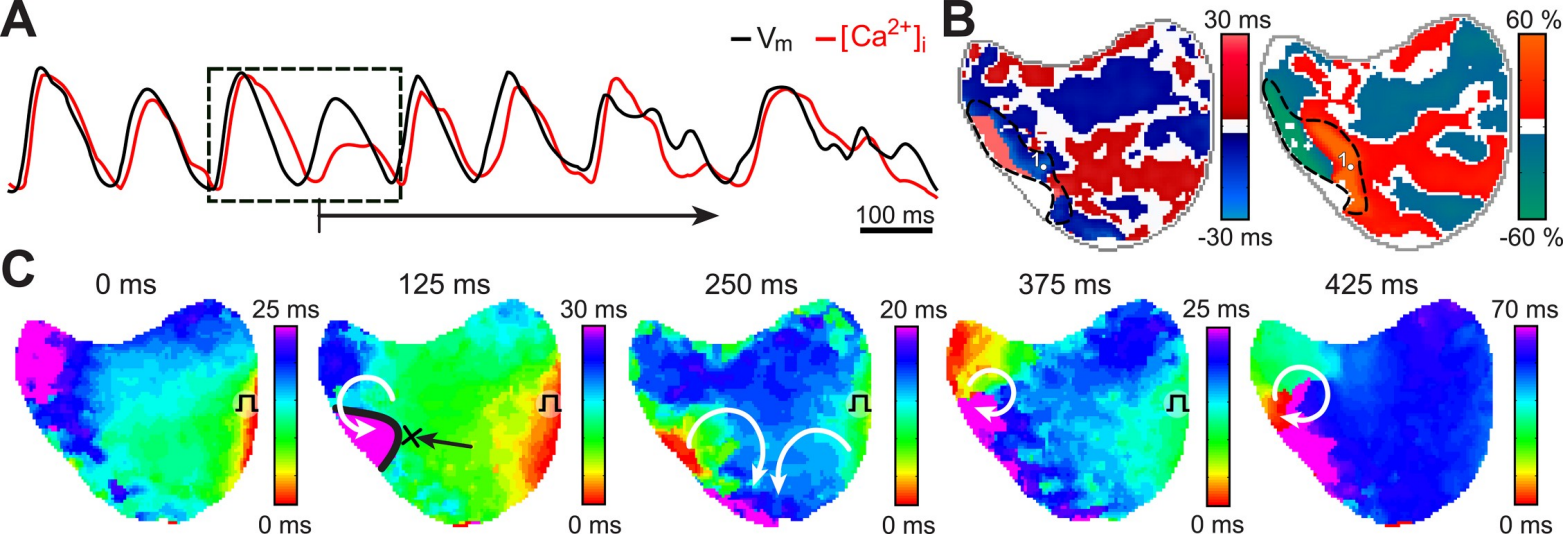
Sequence of VF induction. **(A)** Recordings of optical voltage and calcium signals of induction of VF, belonging to point 1 in panel B. **(B)** Distribution of APD and CaT alternans during two beats previous to the VF onset, the marked shaded line indicates the region with the highest alternans. **(C)** Sequence of isochronal maps corresponding to the traces depicted in panel A. Notice how during the second propagation (i.e. 125ms) a line of block was formed. This line of block allowed the appearance of reentrant patterns in the following isochrones and the perpetuation of the VF. A possible pathway of reentrant pattern is suggested by white arrows.

The previously described protocol that consisted in a gradual increase of the pacing rate allowed the induction of VF in all preparations. Under basal conditions, VF was induced at a stimulation rate of 132.5 ± 16 ms. The precursor mechanism which initiates VF can be observed in a representative example in Fig 3. In Fig 3C, the sequence of isochronal maps depicts the formation of a unidirectional block (i.e. 125 ms). This unidirectional block allowed the appearance of reentrant patterns in the following isochrones and the perpetuation of the VF.

Remarkably, this unidirectional block appears in the area where (i) higher magnitudes of voltage and calcium alternans were observed and (ii) slow conduction occurred (Fig 3B).

In all preparations the same calcium SDA trends were observed before the initiation of VF in the entire mapping area. Despite the difficulties, the specific instant of VF initiation was captured in 3 of the 5 animals. This result suggests that VF initiation was linked with a calcium dynamics mediated phenomenon.

### Effect of verapamil

Previously described results indicate that during our pacing protocol calcium alternans preceded the sequence of events that produced the unidirectional block and reentrant initiation of VF. In order to evaluate the potential effect of modifying the calcium homeostasis, the previously described analysis was repeated in each preparation after the administration of a verapamil (an L-type calcium channel blocker).

**Fig 4:**
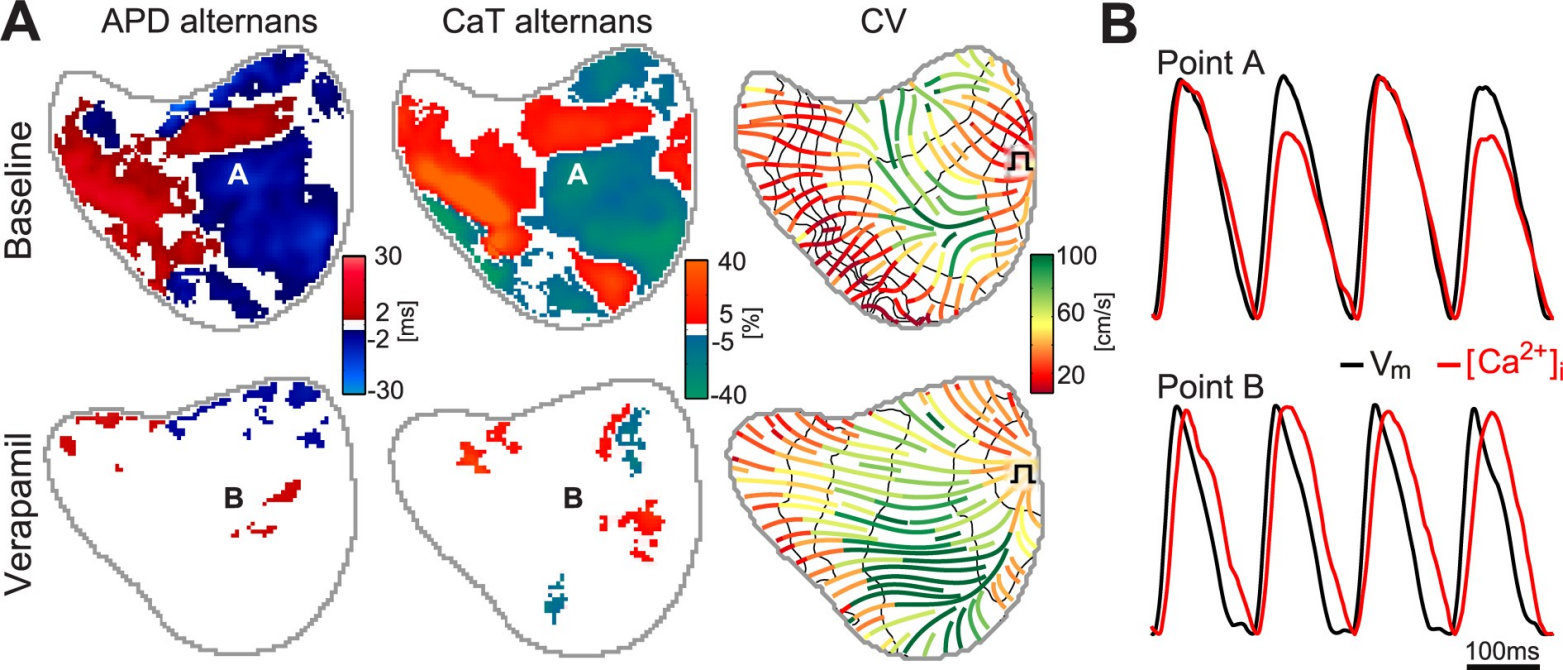
Effect of verapamil administration on alternans and CV. **(A)** Distribution of APD and CaT alternans maps together with the mean local CV at baseline, compared to same maps computed at the same pacing rate after administration of verapamil. **(B)** Normalized voltage and calcium optical signals of points A and B labeled on alternans maps.

In Fig 4A we obtained maps of voltage, calcium alternans and conduction velocity during basal conditions just before VF initiation (i.e. 140ms) and at the same pacing rate after the administration of verapamil. Notice that the existing alternans were not reproduced after drug administration; when verapamil was infused; the total area of alternans was significantly smaller, from a ΔAPD area of 76% to 19% and ΔCaT area of 63% to 17%. Regarding the CV, the changes were not as noticeable. Fig 4B shows the change in morphology of action potentials and calcium transients. Notice that dual V_m_/Ca^2+^ trace demonstrate a signal relationship in which the voltage signal always leads the calcium signal. This was the case in all pixels and activations both during slow and fast pacing rates.

As it can be observed, verapamil produced a significant reduction on the duration of action potentials and a stabilization of calcium transients, without alternans.

**Fig 5:**
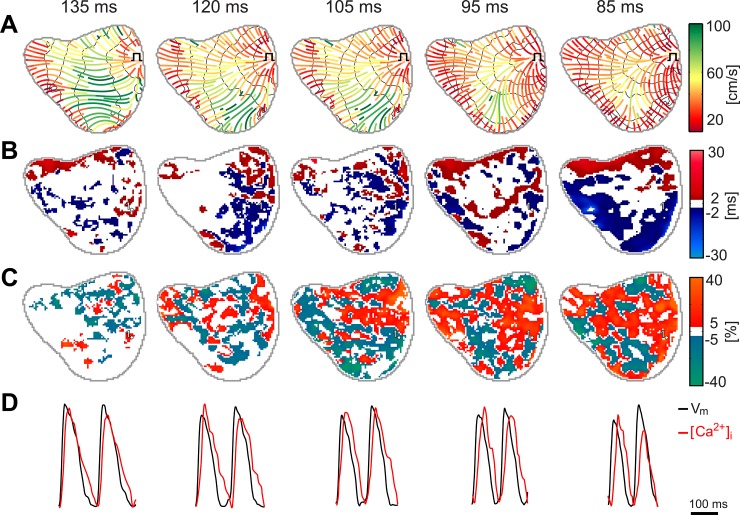
Rate-dependent development of electrophysiological features obtained at different pacing cycle length after verapamil administration. **(A)** Distribution of local CV through the trajectory of propagation over isochrones fragmented in steps of 5 ms (black). **(B)** Maps of APD alternans obtained between successive wavefronts. **(C)** Maps of CaT alternans obtained between successive propagations. **(D)** Recordings of optical voltage and calcium signals.

After the administration of verapamil, all preparations allowed faster stimulation rates before the induction of VF. A representative example of the evolution of SDA on treated preparations is shown in Fig 5. As expected, the increase on pacing rate was associated with a significant reduction in CV. The area covered by voltage and calcium SDA increased for fast activation rates. Interestingly, SDA patterns after the administration of verapamil presented a more patched distribution with a low correspondence between voltage and calcium alternans domains.

### Spatiotemporal mechanisms of VF inducibility

**Fig 6:**
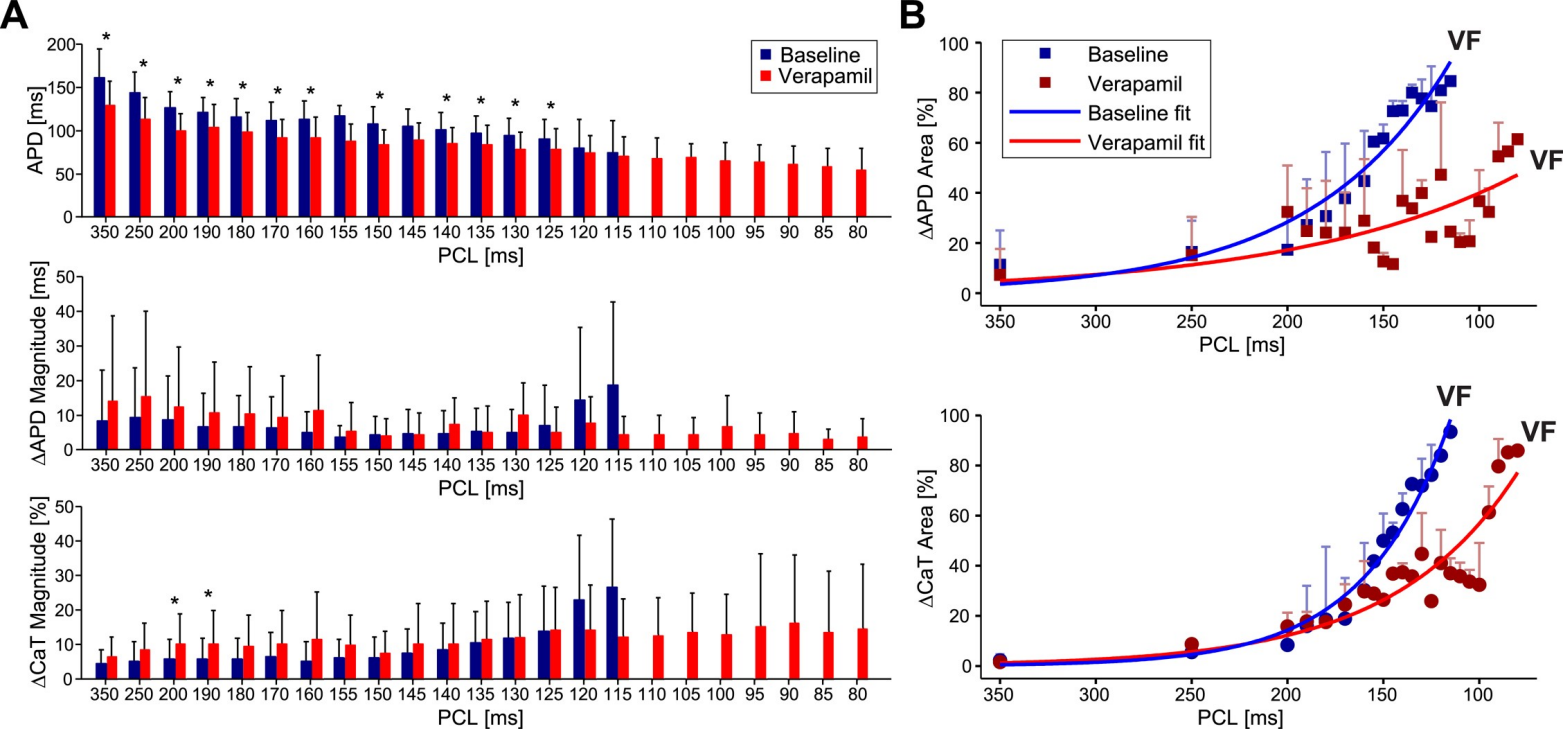
Rate-dependent changes of APD, voltage and calcium alternans for the entire dataset. **(A)** Progression of APD (top panel), magnitude of APD alternans computed (middle panel) and magnitude of CaT alternans (bottom panel). **(B)** Percentage of area of APD (top panel) and CaT alternans (bottom panel) that covers the ventricular surface recorded during the application of the protocol of increasing rhythms during basal conditions and after verapamil administration. * Means p< 0.05.

In order to elucidate the potential role of each electrophysiological parameter on the mechanisms of initiation of VF, we examined the rate-dependent changes of APD, ΔAPD and ΔCaT magnitudes (Fig 6A) and epicardial area covered by them (Fig 6B), both during basal conditions and after the administration of verapamil.

As previously described by Mironov et al. (2008), during basal conditions the increase on activation rates produced a reduction on mean duration of action potentials. It is important to note that this reduction of APD was associated with higher magnitudes of APD alternans only during basal conditions. After the administration of verapamil, the magnitude of APD alternans were independent of the pacing rates. In fact, despite the significant reduction of APD produced by verapamil for slow pacing rates, those differences disappeared for fast pacing rates (i.e. 120ms and 115ms).

Regarding the trends of oscillations of ΔAPD and ΔCaT alternans during basal conditions, magnitude and standard deviation increased gradually with the activation rate until the onset of VF (i.e. blue bars). Administration of verapamil produced a dissociation between voltage and calcium alternans: ΔAPD remained at low magnitudes independently of the pacing rate, whereas ΔCaT magnitude increased following fast rates.

Notice how in all cases in the area covered by voltage or calcium alternans (Fig 6B), faster activation rates were associated with higher percentage of area affected by alternans. During basal conditions VF was induced when both voltage and calcium alternans were covering almost 100% of the mapped tissue. After the administration of verapamil, VF was induced when calcium alternans were covering almost 100% of the mapped tissue, whereas voltage alternans were present on less than 70% of the tissue. These results suggest that, at least after the administration of verapamil, calcium alternans play a main role in the sequence of events that triggers VF.

**Fig 7:**
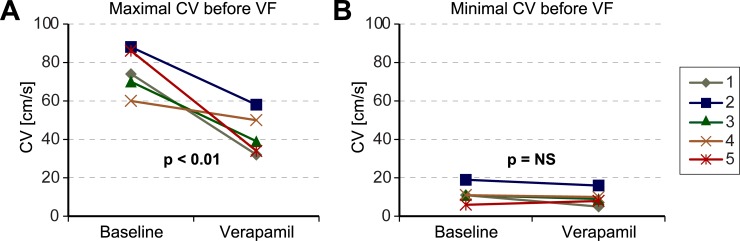
Maximal and minimal CV recorded previously to the induction of VF. **(A)** Maximal and **(B)** minimal CV measured in the optical mapping with the fastest pacing rate previously to the induction of VF, both during basal conditions and after verapamil administration. * Means p< 0.01.

Finally, in order to elucidate the role of CV on triggering VF, the variations of CV just before the initiation of VF were compared during basal conditions and after the administration of verapamil. These results indicate that the administration of verapamil produced a significant reduction of CV (Fig 7A), 74.9 ± 10.4 cm/s vs 41.5 ± 9.9 cm/s, p < 0.01. However, if the areas with slower CV are compared; similar values of minimal CV were observed just before the initiation of VF both during basal and treated conditions, 10.9 ± 4.2 cm/s vs 8.9 ± 3.6 cm/s with not significant differences (Fig 7B).

## Discussion

### Major findings

Major new findings of this study are as follows. First, onset of ventricular fibrillation in a high pacing rate model is linked with the conjunction of spatial calcium alternans and slow CV regions. Second, modification of L-type calcium current by means of verapamil is associated with a dissociation of the evolution between calcium and voltage spatial alternans. These results highlight the relevance of spatial electrophysiological properties such as CV and calcium SDA when a drug is applied.

### Mechanisms of initiation of VF and safety pharmacology

Initiation of cardiac arrhythmias requires the combination of a trigger event and a substrate that could maintain reentry [25]. Initiation of VF has been associated with different mechanisms as for example premature ventricular complexes, caused by delayed afterdepolarizations, rapid triggers emanating from damaged areas such as Purkinje fibers or infarcted tissue [25], or steeply sloped restitution curves that create unstable wave propagation resulting in wave break, the event that is necessary for fibrillation [26]. Despite these different mechanisms, one of the main risk marker commonly used as safety control in pharmaceutical trials is the QT prolongation [27], associated with afterdepolarizations that may produce activation blocks and reentry. From another perspective, the prolongation of action potential duration is also the target of many antiarrhythmic treatments [28,29], since long action potentials may reduce the probability to maintain reentry [30] during or after the appearance of rapid triggers.

These different scenarios perfectly illustrate the complexity of identifying a single *in-vitro* or *in-vivo* test that predicts the potential proarrhythmic effects of a novel drug. Cardiac arrhythmias, and specially VF, can be the result of many different processes. There are many factors that are rhythm and/or spatially dependent on the cardiac physiology, as the appearance of alternans or heterogeneous distributions of ionic expressions. Several experimental and computational studies have shown how initiation of cardiac arrhythmias may be the result of heterogeneous substrate regarding local CV distribution [12,31], voltage instability driven by a steep restitution slope, development of APD [32] or CaT spatially discordant alternans [5,33]. Consequently, the evaluation of safety pharmacology may require mapping technologies that allow the detection of spatial heterogeneities induced by the treatment. Effects of drugs on propagation properties, such as conduction velocity or calcium associated phenomena, which remain difficult to evaluate by means of electrical mapping, may significantly influence the probability of suffering an arrhythmia. In this sense, multiparametric optical mapping has become an extended technique that allows fast and reproducible analysis of several samples, from cells to whole organs, allowing the characterization of spatiotemporal behavior [19].

### Role of calcium alternans and conduction velocities on VF initiation

Several studies have focused on the role of intracellular calcium on the appearance of the development of complex patterns that form a substrate prone to VF [5,34,35], even in absence of ion current abnormalities. Specifically, overload of intracellular calcium in healthy cardiac tissue, caused by pacing rates or pressures out of the physiological limits, may form heterogeneous substrate prone to the generation of VF. The approach of these studies is usually done with isolated cells [35], cardiac cultures [33] or using numerical simulations [11,36–38] while similar studies of intracellular calcium distribution in whole isolated hearts are focused in other investigation lines [12,20,39–41].

In this article, we describe our approach to data processing and parameter extraction to characterize APD and CaT alternans and conduction velocity. Our results on isolated hearts are in accordance with previous studies in which the balance between increased amount of calcium instabilities and lower conduction velocity is a suitable stage prone to arrhythmic triggers [23,42], suggesting that the control of this balance may work as a therapeutic target. Our results show that during basal conditions calcium transient has a local onset similar to APD. Interestingly, this coordination was lost after the administration of verapamil, indicating that different mechanisms could be driving voltage and calcium alternans.

In order to modify the balance between sodium and calcium, we have evaluated the effects of a calcium blocker; verapamil. Different sources have shown the therapeutic effect that administration of verapamil [13,16,43] or other blockers [26] exert on VF modulation, reducing incidence of fibrillatory episodes or reentry termination. The main conviction of its effect is the flattening of restitution curve that this class of drugs produces in the different experimental setups. However, other reports [44] have suggested no direct link between the restitution slope with the appearance of alternans and arrhythmias. Our results indicate that after the administration of verapamil, faster activation rates are needed to produce alternans apparition, maintenance and spreading. This reduction in the alternance was correlated with a lower arrhythmogenicity. In this sequence of events, it has been shown how a reduced incidence of SDA, especially in the range of calcium alternans oscillations combined with an unchanged minimum CV, played a crucial role in reducing the generation of unidirectional blocks and reentry that promote VF.

## Limitations

Our observations are restricted to healthy rabbit hearts and, therefore, extrapolating these results to other models should be done carefully. The use of pathological hearts, with for example chronic infarct hearts, could increase the efficacy of the technology evaluation the risk of novel drugs. CV measurements from optical mapping may be affected by curving of the heart or perpendicular propagation of the wavefront towards the field of view, leading to overestimated CVs. However, presented measurements have been compared between exactly same points of view; consequently CV reductions observed during fast pacing rates or after the administration of the drug are independent of the mentioned limitation.

In this study, we choose to evaluate verapamil due to its controversial electrophysiological properties and its potential effects over alternans [23,43,45]. Nevertheless, optical mapping technology could be used to evaluate the effect of both proarrhythmic and antiarrhytmic drugs. In addition, it is important to realize that existing optical mapping techniques require electromechanical dissociation which may influence calcium homeostasis. Despite of that, this is the only technology that is available nowadays for quantifying calcium dynamics.

## Clinical implications

T wave alternans has been proposed as a clinical biomarker of arrhythmia risk [46] and may be a major factor in the decision to prescribe the use of an implantable defibrillator or antiarrhythmic therapy. Our results indicate that alternans are linked with the induction of VF when they are associated with slow conduction velocities. Consequently, our study emphasizes the need to analyze T-wave alternans together with spatial properties that could serve to estimate the heterogeneities on conduction velocities. In addition, recent studies on porcine models demonstrated that QRS duration may reflect underlying changes in CV during increased intraventricular pressure and heart failure [47]. However, increases of QRS duration may also be related with changes on cell size regardless of CV values [48].

## Conclusions

In the present study, we have shown that the combination of spatially discordant cardiac alternans and heterogeneous CVs is associated with reentry and VF initiation. This sequence of events can be modified by the administration of a drug and therefore change the onset of VF. In addition, the analysis of APD duration may not be sufficient to predict the safety or pro-arrhythmogenicity of a drug.

## Supporting information

S1 Appendix S1_Minimal_Data_Set.xlsx. Data obtained to calculate each parameter.
(XLSX)
